# Assessment of Enzymatically Derived Blackcurrant Extract as Cosmetic Ingredient—Antioxidant Properties Determination and In Vitro Diffusion Study

**DOI:** 10.3390/pharmaceutics16091209

**Published:** 2024-09-14

**Authors:** Anja Petrov Ivanković, Marija Ćorović, Ana Milivojević, Stevan Blagojević, Aleksandra Radulović, Rada Pjanović, Dejan Bezbradica

**Affiliations:** 1 Innovation Centre of Faculty of Technology and Metallurgy, 11000 Belgrade, Serbia; apetrov@tmf.bg.ac.rs; 2 Faculty of Technology and Metallurgy, University of Belgrade, 11000 Belgrade, Serbia; amilivojevic@tmf.bg.ac.rs (A.M.); rada@tmf.bg.ac.rs (R.P.); dbez@tmf.bg.ac.rs (D.B.); 3 Institute of General and Physical Chemistry, University of Belgrade, 11000 Belgrade, Serbia; sblagojevic@iofh.bg.ac.rs (S.B.); aradulovic@iofh.bg.ac.rs (A.R.)

**Keywords:** blackcurrant extract, antioxidant activity, anthocyanins, cosmetic formulation, diffusion, topical delivery

## Abstract

Blackcurrant is an anthocyanin-rich berry with proven antioxidant and photoprotective activity and emerging prebiotic potential, widely applied in cosmetic products. Hereby, highly efficient enzyme-assisted extraction of blackcurrant polyphenols was performed, giving extract with very high antioxidant activity. Obtained extract was characterized in terms of anthocyanin composition, incorporated into three different cosmetic formulations and subjected to Franz cell diffusion study. Experimental values obtained using cellulose acetate membrane for all four dominant anthocyanins (delphinidin 3-glucoside, delphinidin 3-rutinoside, cyanidin 3-glucoside and cyanidin 3-rutinoside) were successfully fitted with the Korsmeyer–Peppas diffusion model. Calculated effective diffusion coefficients were higher for hydrogel compared to oil-in-water cream gel and oil-in-water emulsion, whereas the highest value was determined for cyanidin 3-rutinoside. On the other hand, after a 72 h long experiment with transdermal skin diffusion model (Strat-M^®^ membrane), no anthocyanins were detected in the receptor fluid, and only 0.5% of the initial quantity from the donor compartment was extracted from the membrane itself after experiment with hydrogel. Present study revealed that hydrogel is a suitable carrier system for the topical delivery of blackcurrant anthocyanins, while dermal and transdermal delivery of these molecules is very limited, which implies its applicability for treatments targeting skin surface (i.e., prebiotic, photoprotective).

## 1. Introduction

The skin is the largest human organ, suitable for administration and both topical and systemic delivery of different drugs [1]. Having a barrier function for pathogenic factors (e.g., microorganisms or allergens) and preventing excessive water loss, human skin, particularly its upper layer, stratum corneum, represents limitation for the penetration of desired substances into deeper skin layers or into the body, which are often desired targets for exhibiting their full therapeutic potential [2]. While drugs that are administrated in order to be systemically absorbed need to be transdermally delivered, those targeting different skin conditions should stay on its surface (e.g., UV protection, prebiotic) or delivered into deeper skin layers (e.g., AHR receptor stimulation, collagen synthesis, inflammatory diseases treatment) [3,4,5,6,7,8].The permeation of active substances through the stratum corneum generally depends on the interaction between the skin, active compound and different formulation components, and that interdependence provides possibility for controlled or sustained drug release which is preferred for topical, cutaneous and systemic delivery [1,3,8].

For the laboratory-scale evaluation of active ingredients’ skin permeation and release from topical formulations, Franz diffusion cell experiments are being usually used with different membranes [7]. When carrier systems are examined for drug release, cellulose acetate membrane with larger pore diameter, which does not represent a diffusion barrier [9,10], is commonly applied, while for cutaneous and transdermal delivery studies, animal models are preferred [11,12]. Recently, synthetic Strat-M^®^ membrane was proven as an adequate replacement for biological skin models since, besides having no ethical issues, it gives an advantage of surface uniformity and constant characteristics, thus providing the highest possible reproducibility and reliability of formulation and active ingredients screening data [13,14].

Blackcurrant is a berry fruit rich in phenolic compounds, particularly anthocyanins [15], which are known for their antioxidant activity and photo-protective role against photo-inhibitory and photo-oxidative damage caused by high light [16]. Polyphenols occur in free forms within the berries but also are bound to the cell wall, which complicates their extraction through traditional solvent-based extraction. In order to overcome this problem, the use of carbohydrate-hydrolysing enzymes to break down the cell wall can significantly improve polyphenol yield in a shorter time. Furthermore, enzyme-assisted extraction is conducted under mild conditions, without the use of organic solvents, making it a more advantageous option than solvent-based extraction [15,17]. We have previously proven that blackcurrant extract possesses the potential to regulate skin microbiota composition by positively influencing *Staphylococcus epidermidis* (skin commensal) and *Staphylococcus aureus* (opportunistic pathogen) balance, whereas this prebiotic effect is even more pronounced when enzymatically derived extract is applied [17]. Therefore, our goal was to characterize blackcurrant extract obtained in an enzyme-assisted process in terms of total polyphenol content and different polyphenol classes’ (flavonoid, flavonol, anthocyanin, phenol acid, hydrolysable tannin and condensed tannins) abundance and to correlate them with antioxidant activity of extracts determined by several in vitro methods (DPPH, ABTS, CUPRAC and FRAP). Furthermore, extract was incorporated in several cosmetic formulations in order to evaluate them as anthocyanin carrier systems for topical, cutaneous and/or transdermal delivery by performing diffusion experiments with cellulose acetate and Strat-M^®^ membrane. Obtained results were modelled in order to determine a release mechanism for each individual anthocyanin compound and calculate their effective diffusion coefficients.

## 2. Materials and Methods

### 2.1. Materials

Lyophilised blackcurrant (LBC), variety Ben Nevis, was supplied by Drenovac d.o.o., Arilje, Serbia. The used blackcurrants have perfectly spherical berries with a diameter of ~1 cm and a deep purple colour coming from their full ripeness. For the extraction process, berries were ground in a mill (Retsch MM 400, Retsch GmbH, Haan, Germany) to a powder.

Analytical grade chemicals, including sodium acetate trihydrate, sodium chloride, sodium carbonate, aluminium chloride, iron(III) chloride hexahydrate and iron(II) sulphate, were obtained from Centrohem (Stara Pazova, Serbia). HPLC-grade solvents (methanol and water), trifluoracetic acid (TFA), 2,4,6-tris(2-pyridyl)-s-triazine, caffeic acid and Trolox were purchased from Sigma-Aldrich (Schnelldorf, Germany). Anthocyanin HPLC standards, delphinidin 3-*O*-rutinoside chloride, delphinidin 3-*O*-glucoside chloride, cyanidin 3-*O*-rutinoside chloride and cyanidin 3-*O*-glucoside chloride were purchased from Carl Roth (Karlsruhe, Germany). Gallic acid and quercetin were obtained from Merck (Darmstadt, Germany). Folin–Ciocalteu phenol reagent was obtained from Carlo Erba (Arese, Italy). Glacial acetic acid, 96% (*v*/*v*) ethanol and hydrochloric acid were from Zorka Pharma (Šabac, Serbia). Commercial enzyme preparation Viscozyme^®^ L was obtained from Novozymes (Bagsvaerd, Denmark), while Rohapect^®^ MC was a kind donation from AB Enzymes (Darmstadt, Germany). The formulations were prepared using Aristofex^®^ AVC polymer from Clariant International Ltd. (Basel, Switzerland), gelling agent HeliogelTM from Lucas Meyer Cosmetics (Paris, France), isopropyl myristate and Lanette^®^ O (cetearyl alcohol) from BASF (Ludwigshafen, Germany), and Sabowax FL 65K (glycerol–stearate, PEG-100 stearate) from (Sabo, Levate, Italy). The cellulose acetate and Strat^®^-M membranes were purchased from Whatman (Dassel, Germany) and Merck Millipore (Billerica, MA, USA), respectively.

### 2.2. Methods

#### 2.2.1. Enzyme-Assisted Extraction

Enzyme-assisted extraction of polyphenols from LBC was performed using a mixture of commercial enzyme preparations—Viscozyme^®^ L and Rohapect^®^ MC, in accordance with a procedure previously optimized by our research group [17]. Briefly, LBC and acetate buffer (pH 4.5) were mixed at a 1:10 ratio, and enzyme mixture was added (Viscozyme^®^ L and Rohapect^®^ MC in a 2 to 1 ratio with overall concentration of 0.05 mL/g DM). Extraction took place at 50 °C with shaking at 150 rpm during 60 min, followed by enzyme deactivation (heating at 100 °C for 5 min). All prepared extracts were stored at −20 °C until further use.

#### 2.2.2. Extracts Compositional Analysis

The total polyphenol content in the extracts was assessed using a modified Folin–Ciocalteu method [18]. The results were expressed as milligrams of gallic acid equivalents (GAE) per gram of dry matter (DM) of the LBC. The total flavonoid content in the extracts was evaluated using the aluminium chloride colorimetric method [19]. The results were presented as milligrams of quercetin equivalents (QE) per gram of dry matter (DM) of the LBC. The total anthocyanin content in extracts was obtained by the pH differential method [19]. The final results were expressed as milligrams of cyanidin-3-*O*-glucoside equivalents (cya-3-glu) per gram of dry matter (DM) of the LBC. The total flavonol content in the extracts was determined using the method reported previously [20]. The results were expressed as milligrams of quercetin equivalents (QE) per gram of dry matter (DM) of the LBC. The total phenolic acid content in the extract was quantified using the method with Arnow’s reagent [21]. The results were expressed as milligrams of caffeic acid (CAE) per gram of dry matter (DM) of LBC. Total hydrolysable tannins content in the extract was determined by the potassium iodate solution method [22]. The total amount of tannins in the sample was expressed in milligrams of gallic acid equivalents (GAE) per gram of dry matter (DM) of LBC. Total condensed tannins content in the extract was determined by the *n*-butanol- hydrochloric acid assay [23]. The concentration of condensed tannins was calculated as milligrams of cyanidin equivalents (CyE) per gram of dry matter (DM) of LBC using molar extinction coefficient (34,700 L/(mol·cm) and molar mass (287.28 g/mol) of cyanidin.

#### 2.2.3. Antioxidant Activity Determination

To assess the antioxidant activity of the extracts, several spectrophotometric methods were applied: ferric-reducing antioxidant power (FRAP) method, 2,2-diphenyl-1-picrylhydrazyl (DPPH) radical scavenging activity assay, 2,2-azino-bis-3-ethylbenzothiazoline-6-sulphonic acid (ABTS) radical scavenging assay and cupric ion-reducing antioxidant capacity (CUPRAC) method. All methods were performed according to previously described protocols [18,24,25], and the final results were expressed as micromoles of Trolox equivalent (TE) per gram of dry matter (DM) of LBC.

#### 2.2.4. Formulation Preparation

Hydrogel was prepared as the 2% (*w*/*v*) water solution of commercial polymer Aristoflex^®^ AVC and mixed using an overhead stirrer (Heidolph Hei-TORQUE Value 100, Schwabach, Germany) at 800 rpm for 5 min until gel formation occurred. To make an oil-in-water (O/W) cream gel, 3% (*v*/*v*) of Heliogel^™^ gelling agent and 10% (*v*/*v*) isopropyl myristate were mixed at 500 rpm for 2 min. Then, 87% (*v*/*v*) water was added, and the mixture was stirred at 1000 rpm for 5 min until a cream gel was formed. For preparation of the oil-in-water (O/W) emulsion, firstly, 84% (*v*/*v*) water and a mixture of 5% (*w*/*v*) emulsifier containing PEG-100 stearate and glycerol–stearate (Sabowax FL 65, Sabo), 1% (*w*/*v*) cetearyl alcohol (Lanette-o, Basf), and 10% (*v*/*v*) isopropyl myristate were heated separately to 75 °C in a water bath (Memmert, Schwabach, Germany). After reaching the desired temperature and melting the lipid components, water and the mixture were combined, and stirred at 500 rpm for 1 min, followed by stirring at 1000 rpm for 2 min, and then at 700 rpm for 3 min. After the temperature of the emulsion dropped to 50 °C, it was stirred at 1100 rpm for 1 min to achieve better dispersion of oil particles in the aqueous phase. Blackcurrant extract was added to all formulations to the final concentration of 1% (*v*/*v*).

#### 2.2.5. Release Studies

Experiments were performed using Franz diffusion cell (20 mL volume and 25 mm orifice diameter, PermeGear, Inc., Hellertown, PA, USA) and two membrane types—cellulose acetate membrane and transdermal test diffusion model, Strat-M^®^ membrane (Merck KgaA, Darmstadt, Germany). The prepared formulation, weighing 2 g ± 5%, was placed in the donor compartment. The receptor compartment was filled with the 0.01 M phosphate buffer saline (PBS) pH 7.4, which was continuously stirred with a small magnetic bar (750 rpm) and thermostated (25 °C). Before application, the cellulose acetate membrane was pre-soaked in receptor medium for 30 min, whereas the Strat-M^®^ membrane did not necessitate pre-hydration. At the predefined time, the samples (0.5 mL) were collected from the receptor chamber and immediately replaced with equivalent volume of fresh receptor medium. The amount of anthocyanins that crossed the cellulose acetate and Strat-M^®^ membrane was quantified by the RP-HPLC-UV method. Additionally, the Strat-M^®^ membrane was subjected to ethanol extraction to determine if polyphenol molecules remained in the membrane layers after the transdermal diffusion study. The membrane was cut into small pieces (~1 mm^2^) and mixed with 10 mL of 50% (*v*/*v*) ethanol for 24 h at 25 °C with constant stirring at 150 rpm. The resulting solvent extract was then concentrated using a rotary evaporator (Rotavapor R-210, Buchi, Flawil, Switzerland) and analysed by HPLC.

#### 2.2.6. RP-HPLC-UV Analysis

Quantitative analyses of samples and anthocyanin standards were performed by a Dionex Ultimate 3000 Thermo Scientific (Waltham, MA, USA) high-performance liquid chromatography (HPLC) system and a reverse-phase column (ZORBAX Eclipse Plus C18, 4.6, 150 mm, 5 µm) by a method previously applied for blackcurrant anthocyanins separation [17]. We applied gradient elution with solvent (A) H_2_O:TFA = 100:0.1% and solvent (B) MeOH:TFA = 100:0.1% in the following way: 0–5 min isocratic 5% B, 5–45 min gradient from 5% to 50% B, then 45–55 min isocratic 50% B, 55–55.1 min gradient from 50% to 5% B, 55.1–60 min isocratic 5% B. Eluent flow rate of 1 mL/min was used, and the column was kept at 45 °C. The injection volume of the samples was 50 µL. Detection of anthocyanins from analysed extracts and commercial standards was carried out by a UV detector at 520 nm. Anthocyanin concentrations were calculated based on standard curves obtained with commercial anthocyanin standards. These curves were made by injecting the same volume (50 µL) of a serially diluted stock solution of known concertation into HPLC and plotting peak area against concentration for each anthocyanin. The slopes of standard curves were 0.00125 mmol/L for delphinidin-3-glucoside, 0.00128 mmol/L for delphinidin-3-rutinoside, 0.00157 mmol/L for cyanidin-3-glucoside and 0.00160 mmol/L for cyanidin-3-rutinoside. The retention times were 24.7 min for delphinidin-3-glucoside, 25.9 min for delphinidin-3-rutinoside, 27.0 min for cyanidin-3-glucoside and 28.4 min for cyanidin-3-rutinoside.

#### 2.2.7. Calculation of Effective Diffusion Coefficients

The experimentally obtained results for the initial release time (first 6 h) in this study were modelled using the Korsmeyer–Peppas equation:

(1) $$\frac{M_{t}}{M_{\infty }}=k\cdot t^{n}$$

where M_t_ is an amount of bioactive compound released at time t, M_∞_ is an amount of drug release after infinite time, k is a kinetic constant and *n* is an exponent denoting the characteristic of the release mechanism of the system. In case of the thin film, *n* = 0.5 signifies Fickian diffusion, *n* = 1.0 represents relaxation of the polymeric chain, and the 0.5 ˂ *n* ˂ 1.0 suggests anomalous or non-Fickian diffusion, indicating a combined effect of both Fickian diffusion and polymer relaxation [26]. Additionally, if there is a delay (t_lag_) before the diffusion starts, the modified form of Equation (1) was employed [27,28].

(2) $$\frac{M_{t}}{M_{\infty }}=k\cdot {\left(t-t_{lag}\right)}^{n}$$

Effective diffusion coefficients (D_eff_) were finally calculated as follows [27,28]:

(3) $$D_{eff}=\frac{\pi \cdot \delta^{2}\cdot k^{2}}{4}$$

where *δ* represents the sum of membrane and formulation thicknesses.

The quality of model predictions with respect to the experimental values was evaluated using mean percentage error (MPE):

(4) $$MPE\left(\%\right)=\frac{1}{n}\overset{n}{\underset{i=1}{\sum }}\frac{\left|Mt_{i}^{exp}-Mt_{i}^{mod}\right|}{Mt_{i}^{exp}}\cdot 100$$

where *n* is the total number of measurements for each component being analysed, Mt_i_^exp^ is the amount of the bioactive component as observed in the experiment and Mt_i_^mod^ is amount of bioactive component calculated through the model [29].

#### 2.2.8. Statistical Analysis

All experiments were repeated twice, and the results of experiments were provided as mean ± standard deviation.

## 3. Results and Discussion

### 3.1. Polyphenol Content and Antioxidant Activity of Blackcurrant Extract

The major classes of polyphenols found in blackcurrants include flavonoids, such as anthocyanins and flavonols, as well as phenolic acids and tannins. The combination of these antioxidant compounds in blackcurrants contributes to their numerous health benefits and makes them a valuable component in functional foods and bioactive cosmetics [30]. To assess the polyphenol content and antioxidant properties of enzymatically derived blackcurrant extract, various spectrophotometric assays were employed.

The polyphenolic composition of blackcurrant extract obtained with Viscozyme^®^ L and Rohapect^®^ MC, enzyme preparations with high cellulolytic and pectinolytic activity, are presented in Table 1. The total polyphenol content was 26.9 mg GAE/g DM, which is about 3 times higher compared to the polyphenol content of extract obtained by conventional extraction process (9.35 mg GAE/DM [17]) and ultrasound-assisted extraction (10.23 mg GAE/g DM [31]). Total contents of major polyphenol classes, flavonoids and phenolic acids, were 3.98 mg QE/g DM and 3.85 mg CAE/g DM, respectively. It is known that predominant flavonoids in blackcurrants are anthocyanins, which contribute to their vibrant purple colour and are considered to be particularly potent antioxidants [30]. Their total content in this extract reached 2.21 mg Cy3GE/g DM. Flavonols, another important subclass of flavonoids, were present in extract at a concentration of 2.57 mg QE/g DM, which is significantly higher compared to the previously reported flavonol content in blackcurrant juice (1.31 mg QE/g DM [32]). Both classes of tannins, hydrolysable and condensed, were detected in the extract in concentrations of 12.5 mg GAE/g DM and 0.15 mg CyE/g DM, respectively. This dual presence of hydrolysable and condensed tannins contributes to the diverse and beneficial polyphenolic profile of blackcurrants, showcasing potential antioxidant and health-promoting properties [33].

To evaluate blackcurrant polyphenol’s ability to neutralise free radicals and act as agents in safeguarding cells from oxidative harm, four different methods were applied. These methods were based on single-electron transfer reactions (CUPRAC and FRAP) as well as mixed electron and hydrogen atom transfer (ABTS and DPPH) [34]. The antioxidant activity of blackcurrant extract obtained through enzyme-assisted extraction was 338.4 µmol TE/g DM determined with the CUPRAC method, whereas the FRAP method yielded an antioxidant capacity of 122.6 µmol TE/g DM. This result indicates an increased antioxidant activity compared to results of study using ultrasound-assisted extraction of blackcurrant berries, where the antioxidant activity measured by the FRAP method was 36.5 µmol TE/g DM [31].

According to the ABTS method, the antioxidant activity of blackcurrant extract in the present study was 208.2 µmol TE/g DM, while the value obtained through the DPPH method was 103.9 µmol TE/g DM. For example, the antioxidant activity of blackcurrant berry cultivars previously determined by the ABTS method was up to 54 µmol/TE g DM [35], indicating that enzyme-assisted extraction not only enabled increased extraction efficiency by releasing polyphenols trapped in cell wall structures but further enhanced the extract antioxidant activity. By neutralizing different free radicals, polyphenolic compounds present in enzymatically derived blackcurrant extract showed versatile antioxidant activity. This underscores their potential as effective agents in mitigating damage caused by oxidative stress and promoting overall cellular well-being by both oral and topical administration.

#### Determination of Blackcurrant Anthocyanins’ Composition

**Figure 1:**
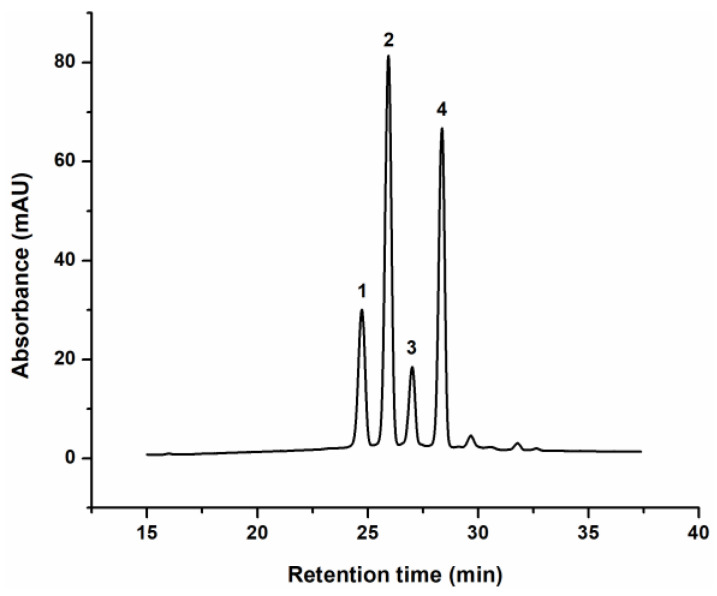
Characteristic chromatogram of the anthocyanins from blackcurrant extract: (1)—delphinidin-3-glucoside, (2)—delphinidin-3-rutinoside, (3)—cyanidin-3-glucoside and (4)—cyanidin-3-rutinoside (cya-3-glu), recorded at 520 nm.

Since anthocyanins are polyphenols of particular interest for cosmetic application, due to their high dermatologic activity and significant role in improving the appearance of the skin, their composition in enzymatically obtained blackcurrant extract was analysed in more details [36]. For that purpose, reverse-phase HPLC-UV analysis was performed, and the characteristic HPLC profile of the extract, recorded at 520 nm, is presented in Figure 1. Based on commercial standards, four major anthocyanin compounds were identified, delphinidin-3-glucoside (del-3-glu), delphinidin-3-rutinoside (del-3-rut), and cyanidin-3-glucoside (cya-3-glu) and cyanidin-3-rutinoside (cya-3-rut), at concentrations of 1.34 mmol/L, 3.43 mmol/L, 0.92 mmol/L and 3.25 mmol/L, respectively. As previously reported, blackcurrant extract anthocyanin composition strongly depends on the extraction method used and plant cultivars [35,37], which determines the share of each molecule in the extract. In order to evaluate the possibility of topical, cutaneous and/or transdermal delivery of identified anthocyanins, enzymatically obtained extract of defined anthocyanin composition was incorporated in different cosmetic formulations, and their permeation through two different membranes was monitored.

### 3.2. Release Study

**Figure 2:**
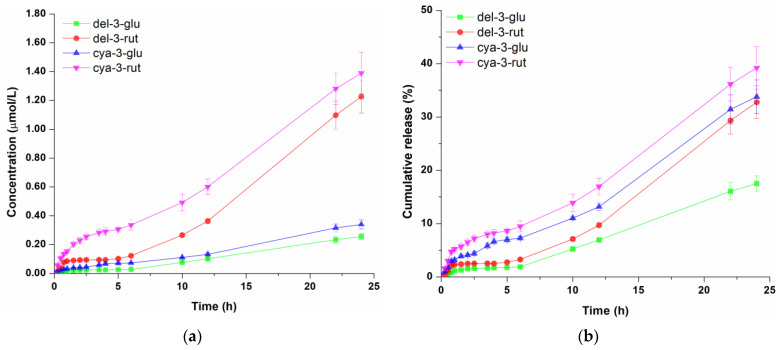
Concentration profile (**a**) and cumulative release (**b**) of anthocyanins from the hydrogel. Data represent the mean values of three independent experiments, and error bars indicate the standard deviations.

**Figure 3:**
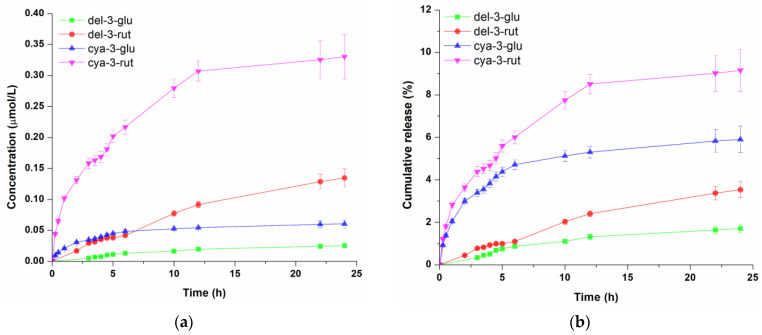
Concentration profile (**a**) and cumulative release (**b**) of anthocyanins from the oil-in-water gel cream. Data represent the mean values of three independent experiments, and error bars indicate the standard deviations.

**Figure 4:**
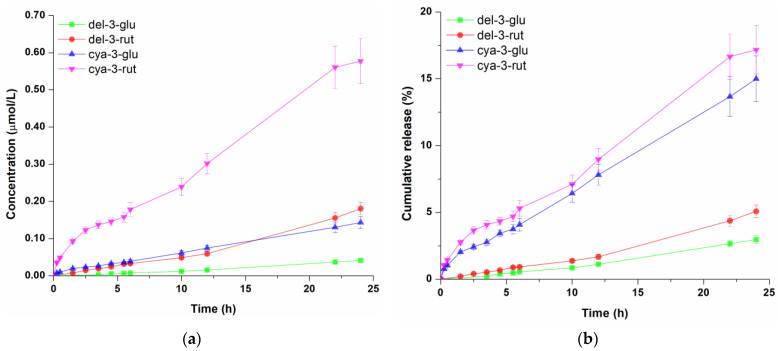
Concentration profile (**a**) and cumulative release (**b**) of anthocyanins from the oil-in-water emulsion. Data represent the mean values of three independent experiments, and error bars indicate the standard deviations.

**Figure A1:**
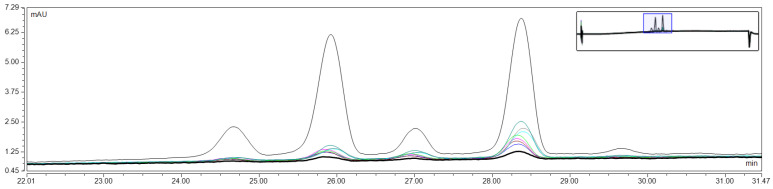
Representative overlapped chromatograms of anthocyanins diffusion from hydrogel over time.

Anthocyanin-rich blackcurrant extract at a concentration of 1% (*v*/*w*) was incorporated into three different cosmetic formulations (hydrogel, oil-in-water cream gel and oil-in-water emulsion), and the diffusion profiles of four anthocyanins across cellulose acetate membrane were determined. Cellulose acetate membrane was applied to assess the performance of different topical formulations to be used as carrier for polyphenol compounds [10]. The molar concentrations of anthocyanins in the receptor compartment which diffused from tested cosmetic formulations through cellulose acetate membrane as well as their cumulative release (the proportion of each diffused anthocyanin) profiles are presented in Figure 2, Figure 3 and Figure 4. Representative overlapped chromatograms of anthocyanins diffusion over time are provided in the Appendix A as Figure A1.

Based on concentration profiles of molecules released from hydrogel shown in Figure 2a, it is obvious that the amount of athocyanidin-3-rutinosides (cya-3-rut and del-3-rut) was significantly higher over time compared to anthocyanidin-3-glucosides (cya-3-glu and del-3-glu).The concentration of cya-3-rut was consistently higher than del-3-rut, with the greatest rise occurring within the initial 2 h of the experiment, after which it displayed a somewhat slower diffusion rate. Since higher initial amounts of del-3-rut were present in the donor compartment compared to cya-3-rut, it can be assumed that differences in their molecular structures are influencing their transmembrane diffusion. On the other hand, the detected concentrations of cya-3-glu and del-3-glu were very similar over the observed period. After 24 h of diffusion, del-3-glu, del-3-rut, cya-3-rut and cya-3-glu molecules reached concentrations of 0.255 µmol/L, 1.226 µmol/L, 0.340 µmol/L and 1.389 µmol/L, respectively, in the receptor compartment. When comparing the cumulative releases (Figure 2b), it can be observed that the release of both cyanidin molecules were very close and constantly higher than the release of both delphinidins molecules. On the other hand, del-3-glu and del-3-rut had similar profiles only in the first 6 h of diffusion, followed by significantly faster release of del-3-rut. The proportion of diffused cyanidin molecules during the first 6 h was 7.26–9.47%, while the proportion of both delphinidin molecules was 1.88–3.27%, indicating slower diffusion of delphinidin glycosides compared to cyanidin glycosides. Notably, after 24 h of diffusion, the proportion of cya-3-rut remained the highest at 39.22%. There was no significant difference in the proportions of cya-3-glu and del-3-rut with cumulative amounts of 32.78% and 33.81%, respectively, while only 17.51% of the initial del-3-glu amount diffused during the observed time, indicating sustained release from Aristoflex^®^ AVC-based hydrogel.

The release kinetic of blackcurrant anthocyanins from the second tested formulation, o/w cream gel, which is shown in Figure 3a, revealed that cya-3-rut was most abundant in the receptor solution (0.330 µmol/L), followed by del-3-rut (0.135 µmol/L), cya-3-glu (0.060 µmol/L) and del-3-glu (0.024 µmol/L). However, the proportion of diffused cya-3-rut during 24 h (Figure 3b) was notably reduced, almost 4.3 times lower than that observed from the hydrogel (9.15%), while the cumulative amount of the other three anthocyanins was even lower—5.7, 9.3 and 10.2 times for cya-3-glu, del-3-rut and del-3-glu, respectively.

When it comes to the diffusion of anthocyanins from the o/w emulsion, the same concentration trend of these compounds was observed (Figure 4a) as with hydrogel and o/w cream gel. Regarding diffused amounts, 0.041 µmol/L, 0.181 µmol/L, 0.143 µmol/L, 0.577 µmol/L of del-3-glu, del-3-rut, cya-3-glu and cya-3-glu were detected in the receptor fluid, respectively. By analysing cumulative amounts of four anthocyanins, it can be seen (Figure 4b) that after 24 h of permeation, the proportions of del-3-glu, del-3-rut, cya-3-glu and cya-3-rut were 3.0%, 5.1%, 15.0% and 17.2%, respectively. These proportions were considerably lower compared to those observed for hydrogel, but slightly higher than those obtained for the o/w gel cream, indicating that reduction of detected molecule concentration in the receptor solution could be ascribed to formulation oil content. Lipid components evidently had a negative effect on the release of the anthocyanins from the o/w cream gel as well as o/w emulsion, which is in line with other studies which showed that the higher share of the oil phase in the formulations led to the slower release of polyphenols [38,39]. A second potential explanation for the faster anthocyanins permeation from the hydrogel compared to oil-based formulations is that these molecules have a sugar moiety in their structure, which gives them an amphiphilic character, making them surface-active. Consequently, it is likely that anthocyanins are more strongly attached to the oil–water interface, hindering their release [39].

#### Determination of Diffusion Coefficients

To comprehensively describe the release profiles of blackcurrant anthocyanins from hydrogel, o/w cream gel and o/w emulsion and find out the mechanism of release as well as kinetic constants, the diffusion data were fitted to the Korsmeyer–Peppas equation (Equation (1)). This equation was suggested by several authors for investigating the release of polyphenols from semisolid formulations [39,40,41]. Effective diffusion coefficients (D_eff_) and kinetic constants of del-3-glu, del-3-rut, cya-3-glu and cya-3-rut from blackcurrant extract incorporated in three formulations across the cellulose acetate membrane are presented in Table 2.

Based on the coefficient of determination (R^2^) and mean percentage error (MPE), it can be confirmed that the chosen release model was suitable for describing the release kinetic of blackcurrant anthocyanins from tested formulations. The obtained value for *n* was 0.5 for all tested formulations, suggesting that the release mechanism of anthocyanins from all formulations was of a Fickian type, highly dependent on molecular diffusion. The lag phase, time before molecules were found in the receptor fluid, was only detected for formulations containing oil and for both delphinidin glycosides, while in other cases, diffusion started immediately after experiment start. As can be seen from Table 2, kinetic constant and, consequently, diffusion coefficients showed trends which are in line with previously discussed results of four anthocyanins’ release profiles and their cumulative amounts. The effective diffusion coefficients for del-3-glu, del-3-rut, cya-3-glu and cya-3-rut permeated from hydrogel were 0.72∙10^−^^8^ cm^2^/s, 1.93∙10^−^^8^ cm^2^/s, 5.46∙10^−^^8^ cm^2^/s and 9.73∙10^−^^8^ cm^2^/s, respectively. In contrast, the D_eff_ of the same molecules released from oil-based formulation were considerably lower compared to water-based hydrogel, even up to ten times.

Although it is known that the molecular weight of molecules typically demonstrates an inverse correlation with their membrane permeability [28,40,42], present study revealed that del-3-rut (611.53 g/mol) and cya-3-rut (595.53 g/mol) had a higher diffusion coefficient from all formulations compared to del-3-glu (465.4 g/mol) and cya-3-glu (449.4 g/mol). This can be attributed to the notably higher initial concentration of rutinose—forms of anthocyanins (disaccharide sugar moiety) in the extract comparing to monoglycosilated ones (Figure 1), thus providing a greater driving force for diffusion [40]. Furthermore, the observed discrepancy in results could potentially be a consequence of variations in the (octanol–water) partition coefficients of molecules, since these coefficients play an important role in the migration of molecules from the formulation to the membrane [39,40,43]. To check the hypothesis, octanol–water partition coefficients (log P) were calculated using Molinspiration calculator (https://www.molinspiration.com/cgi/properties, accessed on 16 July 2024), and their values were −2.99, −3.08, −3.49 and −3.78 for cya-3-glu, del-3-glu, cya-3-rut and del-3-rut, respectively. According to log *p* values, all anthocyanins display hydrophilic characteristics, with those containing rutinose moiety exhibiting slightly greater hydrophilicity, thereby contributing to their enhanced migration across the hydrophilic membrane compared to corresponding molecules with glucose moiety. Additionally, the presence of the delay in the diffusion (t_lag_) of del-3-glu and del-3-rut from both oil-based formulations might be due to their somewhat better solubility in the oil phase because they had to overcome the oil–water interface before the diffusion across membrane [39].

### 3.3. Transdermal Permeation Study

In order to evaluate transdermal permeation of the blackcurrant extract anthocyanins, the Franc diffusion cell with the Strat-M^®^ membrane was employed over a period of 72 h. The analysis of the receptor solution showed that even after this extended permeation process, anthocyanins did not diffuse through the membrane from any of three tested formulations. Given that Strat-M^®^ membrane was specifically engineered to imitate the layered structure and lipid composition found in human skin [44], it was not surprising that anthocyanins, as hydrophilic compounds, would face challenges to permeate through it. A previous study also reported the very weak diffusion of cya-3-glu and del-3-glu from *Vitis vinifera* L. leaf extract through human epidermis, reaching only 0.050% to 0.071% of the initial dose [45]. In order to determine whether a certain percentage of anthocyanins remained in the Strat-M^®^ membrane, the extraction of membrane was performed. The analysis showed that only 0.5% of the initial overall anthocyanin quantity from the donor compartment was extracted from the membrane itself in case of hydrogel, while there were no detectable amounts of anthocyanins in the case of o/w cream gel and emulsion. Such results indicate that tested formulations could provide exclusively topical delivery of blackcurrant anthocyanins, making them suitable for photoprotective or prebiotic skin care.

## 4. Conclusions

The blackcurrant extract obtained with the mixture of Viscozyme^®^ L and Rohapect^®^ MC enzyme preparations showed a high concentration of diverse polyphenolic compounds and high antioxidant activity. This extract was successfully incorporated in three different cosmetic formulations (hydrogel, o/w cream gel and o/w emulsion) from which diffusion of four major anthocyanin molecules (del-3-glu, del-3-rut, cya-3-glu, cya-3-rut) was monitored. In vitro diffusion studies performed with a cellulose acetate membrane revealed that the release of blackcurrant anthocyanins from the tested cosmetic formulations can be adequately described by the Korsmeyer–Peppas diffusion model. Among these formulations, the hydrogel, with no oil content, proved to be the most effective for the release of blackcurrant anthocyanins. Furthermore, anthocyanins with a rutinose moiety had a higher effective diffusion coefficient compared to their corresponding glucoside forms from all formulations. On the other hand, the diffusion across the Strat-M^®^ membrane showed that these molecules are unable to permeate the skin layers, indicating their retention on the skin surface which makes them suitable for the applications targeting upper skin layers such as UV-protective or prebiotic effect. Subsequent research endeavours will focus on refining the formulation and optimizing the composition to enhance the dermal delivery of blackcurrant polyphenols, aiming to broaden their range of applications.

## Figures and Tables

**Table 1:** Polyphenol composition and antioxidant activity of blackcurrant extract.

**Polyphenol Composition**						
**Polyphenols** **(mg GAE/g DM)**	**Flavonoids** **(mg QE/g DM)**	**Anthocyanins** **(mg Cy3GE/g DM)**	**Flavonols** **(mg QE/g DM)**	**Phenol Acids** **(mg CAE/g DM)**	**Hydrolysable Tannins** **(mg GAE/g DM)**	**Condensed** **Tannins** **(mg CyE/g DM)**
26.9 ± 1.45	3.98 ± 0.24	2.21 ± 0.08	2.57 ± 0.13	3.85 ± 0.27	12.5 ± 0.42	0.15 ± 0.01
**Antioxidant activity**						
**FRAP** **(µmol TE/g DM)**		**ABTS** **(µmol TE/g DM)**	**CUPPRAC** **(µmol TE/g DM)**		**DPPH** **(µmol TE/g DM)**	
122.6 ± 7.05		208.2 ± 5.34	338.4 ± 16.64		103.9 ± 5.19	

The results were provided as mean ± standard deviation. The experiments were conducted in duplicate.

**Table 2:** Effective diffusion coefficients and kinetic constants of blackcurrant anthocyanins incorporated in different cosmetic formulations.

Formulation	Compound	t_lag_ (min)	k∙10^4^ (s^−0.5^)	R^2^	D_eff_∙10^8^ (cm^2^/s)	MPE (%)
Hydrogel	del-3-glu	15.0	2.08	0.981	0.72	3.53
	del-3-rut	13.8	3.41	0.960	1.93	6.40
	cya-3-glu	3.6	5.73	0.968	5.47	6.26
	cya-3-rut	0	7.66	0.964	9.73	5.46
O/W cream gel	del-3-glu	150.8	0.87	0.996	0.13	1.95
	del-3-rut	73.4	0.98	0.951	0.17	3.96
	cya-3-glu	0	3.61	0.994	2.26	2.58
	cya-3-rut	0	4.54	0.987	3.57	3.94
O/W emulsion	del-3-glu	180.8	0.60	0.983	0.05	3.18
	del-3-rut	77.4	0.75	0.963	0.08	8.68
	cya-3-glu	3.1	2.98	0.990	1.35	4.10
	cya-3-rut	0	3.93	0.985	2.35	4.13

The results were provided as mean ± standard deviation. The experiments were conducted in duplicate.

## Data Availability

The original contributions presented in the study are included in the article, further inquiries can be directed to the corresponding author.
